# The prognostic comparison among unilateral, bilateral, synchronous bilateral, and metachronous bilateral breast cancer: A meta‐analysis of studies from recent decade (2008‐2018)

**DOI:** 10.1002/cam4.2198

**Published:** 2019-04-30

**Authors:** Bo Pan, Ying Xu, Yi‐Dong Zhou, Ru Yao, Huan‐Wen Wu, Qing‐Li Zhu, Chang‐Jun Wang, Feng Mao, Yan Lin, Song‐Jie Shen, Qiang Sun

**Affiliations:** ^1^ Department of Breast Surgery Peking Union Medical College Hospital, Chinese Academy of Medical Sciences & Peking Union Medical College Beijing P. R. China; ^2^ Department of Pathology Peking Union Medical College Hospital, Chinese Academy of Medical Sciences & Peking Union Medical College Beijing P. R. China; ^3^ Department of Ultrasound Peking Union Medical College Hospital, Chinese Academy of Medical Sciences & Peking Union Medical College Beijing P. R. China

**Keywords:** bilateral breast cancer, metachronous, survival, synchronous, unilateral

## Abstract

**Background:**

The incidence of bilateral breast cancer (BBC) is increasing nowadays comprising 2%‐11% of all breast cancer (BC). According to the interval time between the first and second cancer, BBC could be divided into synchronous (SBBC) and metachronous (MBBC). However, this interval time is quite different across studies. It remains controversial whether the survival of BBC, SBBC, and MBBC is similar or worse compared to that of unilateral breast cancer (UBC), and whether the survival of SBBC is similar or worse compared to MBBC. To better understand the survival of UBC, BBC, SBBC, and MBBC and how the interval time would influence the prognosis of SBBC and MBBC, we performed this meta‐analysis on studies from recent 10 years (2008‐2018).

**Methods:**

Databases of PubMed, Embase, and Web of Science were searched for relevant studies within recent 10 years. Hazard ratio (HR) was adopted to evaluate the difference of overall survival (OS) of UBC, BBC, SBBC, and MBBC. HR of OS comparisons were performed between BBC vs UBC, SBBC vs UBC, MBBC vs UBC, and SBBC vs MBBC with 3, 6, 12 months as the interval time, respectively.

**Results:**

There were 15 studies of 72 302 UBC and 2912 BBC included in the meta‐analysis. The summary HR of OS comparison between BBC vs UBC was 1.68 (95% CI: 1.28‐2.20), SBBC vs UBC was 2.01 (95% CI: 1.14‐3.55), MBBC vs UBC was 3.22 (95% CI: 0.75‐13.78). When 3, 6, 12 months were used as the interval time, the summary HR of the OS comparison between of SBBC vs MBBC were 0.64 (95% CI: 0.44‐0.94), 1.17 (95% CI: 0.84‐1.63) and 1.45 (95% CI: 1.10‐1.92), respectively.

**Conclusion:**

BBC and SBBC showed worse prognosis in terms of OS compared to UBC while MBBC manifested similar or non‐superior survival as UBC. The OS comparison between SBBC and MBBC changed with different interval time used. The longer the interval time used, the worse the survival of SBBC. SBBC with interval of 3‐12 months between the two cancers had the worst prognosis. When 6 months was used to differentiate SBBC from MBBC, these two clinical entities showed similar OS.

## 1 INTRODUCTION

Breast cancer (BC) is the most commonly diagnosed female malignancy worldwide.^1^ The increasing breast cancer incidence rates, improved treatment and growing life expectancy have resulted in the increasing incidence of developing bilateral breast cancer (BBC).^2^ Bilateral breast cancer (BBC) comprised of about 2%‐11% all BC.^2^, ^3^, ^4^ The cumulative incidence rate of developing contralateral BC at 10 years was about 3.4% for unilateral breast cancer (UBC) patients,^5^ and 13%‐40% for women with a BRCA mutation.^6^ Whether the development of BBC compromised the prognosis remained controversial. Studies reported that the prognosis of BBC patients was similar or worse than UBC counterparts.^7^, ^8^, ^9^, ^10^, ^11^, ^12^, ^13^, ^14^, ^15^

According to the interval time between the diagnosis of first and second tumor, BBC can be divided into synchronous (SBBC) and metachronous (MBBC). However, this interval time was quite different among studies and SBBC had been variedly defined as two tumors diagnosed with an interval of 1 month,^16^ 2 months,^17^ 3 months,^7^, ^8^, ^12^, ^14^, ^18^ 6 months^19^, ^20^, ^21^, ^22^ or 1 year.^13^, ^23^, ^24^, ^25^ There were conflicting results among prospective studies^7^, ^8^, ^12^, ^18^ and retrospective studies^10^, ^20^ concerning survival comparison between SBBC vs UBC. Meanwhile, data regarding prognosis of MBBC vs UBC was very sparse. It was unclear how interval time would influence the prognosis of SBBC vs MBBC, although there was evidence that survival of BBC patients differs according to the interval time.^2^, ^4^, ^26^

Clinical practice of breast cancer diagnosis and treatment had been largely improved within the recent 10 years. To better understand the prognostic outcome of BBC, there were three questions to be answered: (1) Whether BBC, SBBC and MBBC would show worse survival than UBC (BBC vs UBC, SBBC vs UBC and MBBC vs UBC)? (2) Whether SBBC would manifest worse prognosis than MBBC, and whether the result of this comparison would be different when the interval time changes (SBBC vs MBBC)? (3) Which group of BBC patients suffered from the worst prognosis? Which interval time would be the most reasonable for clinical practice? To address these questions, we performed this meta‐analysis with available studies only from the recent 10 years (2008‐2018).

## 2 MATERIALS AND METHODS

### 2.1 Data sources and search strategy

The following databases had been searched for relevant studies: PubMed, Embase (OVID) and Web of Science (from 2008 to December 2018). The following medical subject headings and keywords were used for the search: “Breast Neoplasm,” “Breast cancer,” “Bilateral.” The language of literature was restricted to English. Reference lists of all the relevant articles were manually screened by two independent reviewers to ensure the sensitivity of literature search.

### 2.2 Selection criteria and quality assessment

We have used the following inclusion criteria: Articles should be clinical trials, cohort study or case‐control study with full text about the BBC with prognostic data in a specific population, region or country. The following information was extracted: study type, study location, sample size, age, mean follow‐up duration, hazard ratio (HR) of overall survival (OS) with corresponding 95% confidence interval (CI). HR of disease free survival (DFS) was not compared due to insufficient data. We used the Newcastle‐Ottawa quality assessment scale (NOS) to assess the quality of identified studies. Only studies with NOS >5 which were regarded as high‐quality were included. Disparity was resolved by consensus discussion between the two reviewers or by consultation with the third reviewer.

### 2.3 Data extraction

Data had been collected using a predesigned data extraction form by two reviewers. Survival data including HR with confidence interval (CI) and *P*‐value was extracted from the tables or texts of included studies.

### 2.4 Statistical analysis

In this meta‐analysis, hazard ratio (HR) was adopted to evaluate the survival difference between BBC and UBC, as well as between SBBC and MBBC. We used time‐to‐event‐analyses to obtain HRs and associated statistics by carefully manipulating published data when in the absence of individual patient data.^27^ We used fixed effects model to generate the effects for studies without significant heterogeneity (SBBC vs MBBC) whereas random effects model among studies with heterogeneity (BBC vs UBC, SBBC vs UBC and MBBC vs UBC).

I^2^ statistic and the Q statistic had been calculated to evaluate the heterogeneity across studies. Cochran's Q test with *P* < 0.05 or I^2^>50% indicated that included studies had significant heterogeneity. Symmetry of funnel plot was used to assess the publication bias. All analyses were conducted using the Review Manager (RevMan) (Version 5.3. Copenhagen: The Nordic Cochrane Centre, the Cochrane Collaboration, 2014). A value of *P* < 0.05 was considered statistically significant. All statistical tests were two‐sided.

## 3 RESULTS

### 3.1 Characteristics of the included studies

**Figure 1:**
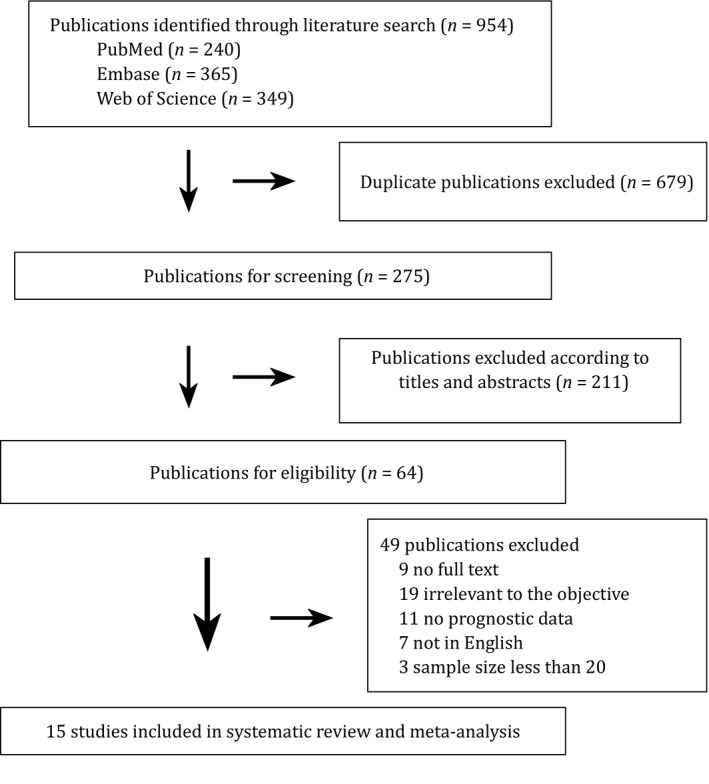
Flowchart of selecting eligible studies for the meta‐analysis

The process and results of studies’ selection were shown in Figure 1. There were totally 954 articles identified, including 240 articles from the PubMed, 365 articles the Embase, and 349 articles from the Web of Science. 275 studies remained after exclusion of duplicates. We obtained 64 potentially relevant studies by sifting through the titles and abstracts. Another 49 studies were excluded for detailed reasons after full‐text reviewing (Figure 1). Consequently, 15 studies were included and the characteristics of these studies were summarized in Table 1.

**Table 1:** Characteristics of studies included in the meta‐analysis

Authors years [reference]	Study location and territory	Publication year (study period)	Study design	Diagnostic interval and sample size	Follow‐up (year)	Overall survival (OS) (y = year, m = month)		HR (95% CI)
Baykara et al^19^	Turkey	2012 (2000‐2011)	Retrospective	<6 m SBBC (n = 60)	3.1	5 y 90%	10 y 81%	SBBC vs MBBC 0.68 (0.34‐1.37)
				>6 m MBBC (n = 90)	9.4	5 y 90%	10 y 73%	
Beckmann et al^7^	Australia	2011 (1997‐2009)	Prospective	<3 m SBBC (n = 52)	6.2	5 y 87.7% (*P* < 0.001)		BBC vs UBC 3.04 (1.82‐5.08)
				>3 m MBBC (n = 35)	6.9	5 y 79.3% (*P* < 0.001)		SBBC vs UBC 1.60 (0.64‐3.86)
				UBC (n = 2336)	5.8	5 y 93.7%		MBBC vs UBC 3.56 (1.54‐8.17)
								SBBC vs MBBC 0.56 (0.24‐1.38)
Ibrahim et al^23^	Egypt	2015 (2005‐2009)	Retrospective	<12 m SBBC (n = 49)	7.2	5 y 60% (*P* = 0.02)		SBBC vs MBBC 1.96 (0.98‐3.92)
				>12 m MBBC (n = 61)	13.3	5 y 78.7% (*P* = 0.02)		
Jobsen et al^8^	The Nertherlands	2015 (1983‐2011)	Prospective	<3 m SBBC (n = 41)	7.2	15 y 31.4% (*P* < 0.001)		MBBC vs UBC 0.60 (0.40‐0.80)
				>3 m MBBC (n = 282)	13.3	15 y 75.0% (*P* < 0.001)		SBBC vs UBC 2.30 (1.50‐3.60)
				UBC (n = 3702)	9.2	15 y 64.3%		BBC vs UBC 1.13 (0.84‐1.52)
Kheirelseid et al^9^	Ireland	2011 (1998‐2008)	Prospective	<12 m SBBC (n = 52)	20.0	Median OS 62 m		BBC vs UBC 1.30 (1.00‐1.70)
				>12 m MBBC (n = 60)		148 m		SBBC vs MBBC 1.16 (0.69‐1.95)
				UBC (n = 2524)		154 m		
Kuo et al^10^	Taiwan	2009 (1990‐1999)	Retrospective	BBC (n = 43)	7.9	15 y 48.8%		SBBC vs UBC 2.91 (0.40‐21.46)
				UBC (n = l,863)	6.6	15 y 68.4%		MBBC vs UBC 8.35 (2.99‐23.32)
								BBC vs UBC 3.27 (2.15‐4.97)
Liang et al^24^	China	2013 (1985‐2010)	Retrospective	<12 m SBBC (n = 84)	3.5	—		SBBC vs MBBC 1.3 (0.79‐2.21)
					8.1			
Nichol et al^11^	Canada	2011 (1989‐2000)	Case‐control	<2 m SBBC (n = 207)	10.2	10 y 71% (*P* < 0.01)		SBBC vs UBC 3.91 (2.93‐5.22)
				UBC (n = 15 497)		l0 y 81% (*P* < 0.01)		
O'Brien et al^28^	USA	2015 (1999‐2007)	retrospective	<3 m SBBC (n = 119)	6.9	5 y 87%	10 y 77%	SBBC vs MBBC 1.05 (0.58‐1.89)
				>6 m MBBC (n = 84)	7.5	5 y 86%	10 y 78%	
Roder et al^12^	Australia and New Zealand	2012 (1998‐2007)	Prospective	<3 m SBBC (n = 813)	4.8	—		SBBC vs UBC 1.17 (0.91‐1.51)
				UBC(n = 34 557)				
Schmid et al^18^	Switzerland	2011 (1990‐2009)	prospective	<3 m SBBC (n = 34)	8.2	5 y 89.4%	10 y 81.7%	SBBC vs UBC 0.93 (0.32‐1.07)
				UBC (n = 100)		5 y 93.8%	10 y 75%	
Schwentner et al^13^	Germany	2012 (NA‐2011)	Retrospective	<12 m SBBC (n = 169) >12 m MBBC (n = 60) UBC (n = 5063)	3.3	—		BBC vs UBC 1.55 (1.15‐2.07) SBBC vs MBBC 1.84 (1.02‐3.32)
Shi et al^14^	China	2012 (2000‐2007)	Retrospective	<3 m SBBC (n = 31)	4.6	5 y 82% (*P* = 0.032)		BBC vs UBC 1.43 (1.07‐1.92)
				>3 m MBBC (n = 106)		5 y 68% (*P* = 0.032)		SBBC vs UBC 4.304 (0.79‐23.47)
				UBC (n = 4046)		5 y 72%		MBBC vs UBC 6.834 (3.63‐12.87)
								SBBC vs MBBC 0.42 (0.24‐0.75)
Wadasadawala et al^22^	India	2018 (2004‐2014)	Retrospective	<6 m SBBC (n = 131)	3.8	3 y 88% (*P* = 0.76)		SBBC vs MBBC 1.67 (1.02‐2.72)
				>6 m MBBC (n = 62)	2.9	3 y 90% (*P* = 0.76)		
Xing et al^15^	China	2015 (2005‐2008)	Retrospective	BBC (n = 81)	4.1	5 y 70.1% (*P* = 0.004)		BBC vs UBC 2.27 (1.54‐3.37)
				UBC (n = 2614)		5 y 87.1% (*P* = 0.004)		

Among the 15 included studies, five studies were from Europe,^8^, ^9^, ^13^, ^18^, ^19^ two from North America,^11^, ^28^ five from Asia,^10^, ^14^, ^15^, ^22^, ^24^ one from Africa,^23^ and two from Australia.^7^, ^12^ The sample size of included studies ranged from 110 to 34 557 and totally 75 214 participants were enrolled, including 72 302 UBC and 2912 BBC (1842 SBBC, 946 MBBC and 124 BBC with unknown interval). The follow‐up time varied from 2.9 to 20 years, with median follow‐up time of 5.8 years (Table 1). There were nine retrospective studies with 13 586 UBC and 1336 BBC cases (median follow‐up time 4.08 years) as well as five prospective studies and one case‐control study with 58 716 UBC and 1576 BBC cases (median follow‐up time 9.17 years) (Table 1).

### 3.2 The comparison of HR of OS between BBC vs UBC

**Figure 2:**
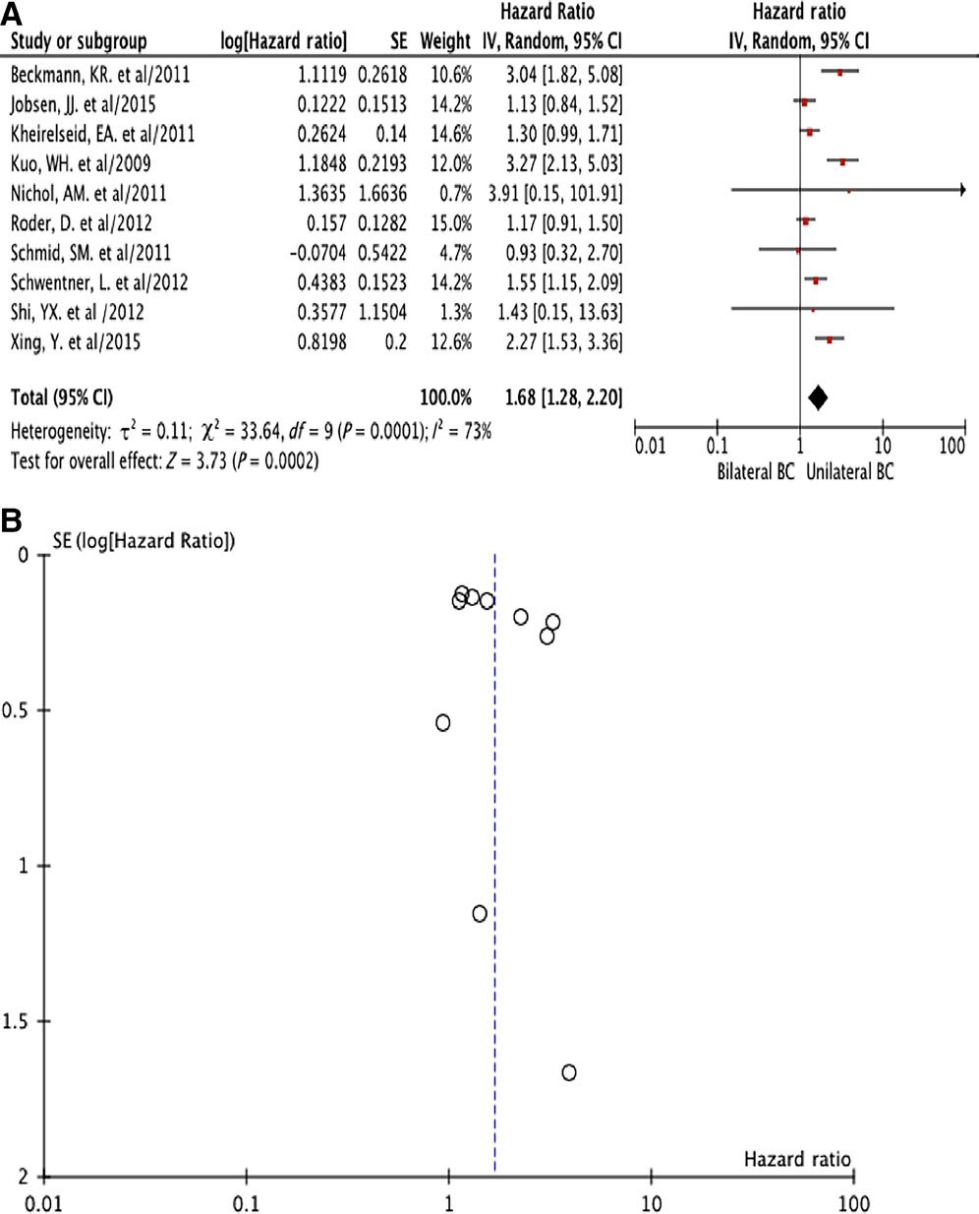
Hazard ratio (HR) of overall survival (OS) comparison of bilateral breast cancer (BBC) vs unilateral breast cancer (UBC): forest plot (A) and funnel plot (B)

There were 10 studies enrolled in the comparison of HR of OS between BBC and UBC, including 2066 BBC cases and 72 302 UBC (Figure 2). The follow‐up time ranged from 3.3 to 20 years, with median follow‐up time 6.6 years. The summary HR of BBC vs UBC was 1.68 (95% CI = 1.28‐2.20) with heterogeneity (I^2 ^= 73%, *P* = 0.0001). So we use random effects model in the analysis. BBC might have worse OS than UBC (Figure 2A).

Sensitivity analysis indicated that the different follow‐up time might be the potential causes of heterogeneity. Thus we divided these studies into three groups by the follow‐up time. In the four studies in which patients were followed <5 years,^12^, ^13^, ^14^, ^15^ the summary HR was 1.46 (95% CI = 1.23‐1.74) without heterogeneity (I^2^ =63%, *P* = 0.05). There were both three studies which followed the cases for 5‐10 years^7^, ^10^, ^18^ and >10 years.^8^, ^9^, ^11^ The summary HR of these two group of studies were 2.85 (95% CI = 2.08‐3.90) and 1.22 (95% CI = 1.00‐1.50) respectively, and there was no heterogeneity in both groups (I^2^ =57%, *P* = 0.10 and I^2^ =0%, *P* = 0.62). These results were all analyzed with fixed effects models respectively and consistent with the results from 10 studies with random effects models that BBC showed worse OS than UBC (Figure 2B).

### 3.3 The comparison of HR of OS between SBBC vs UBC and MBBC vs UBC

**Figure 3:**
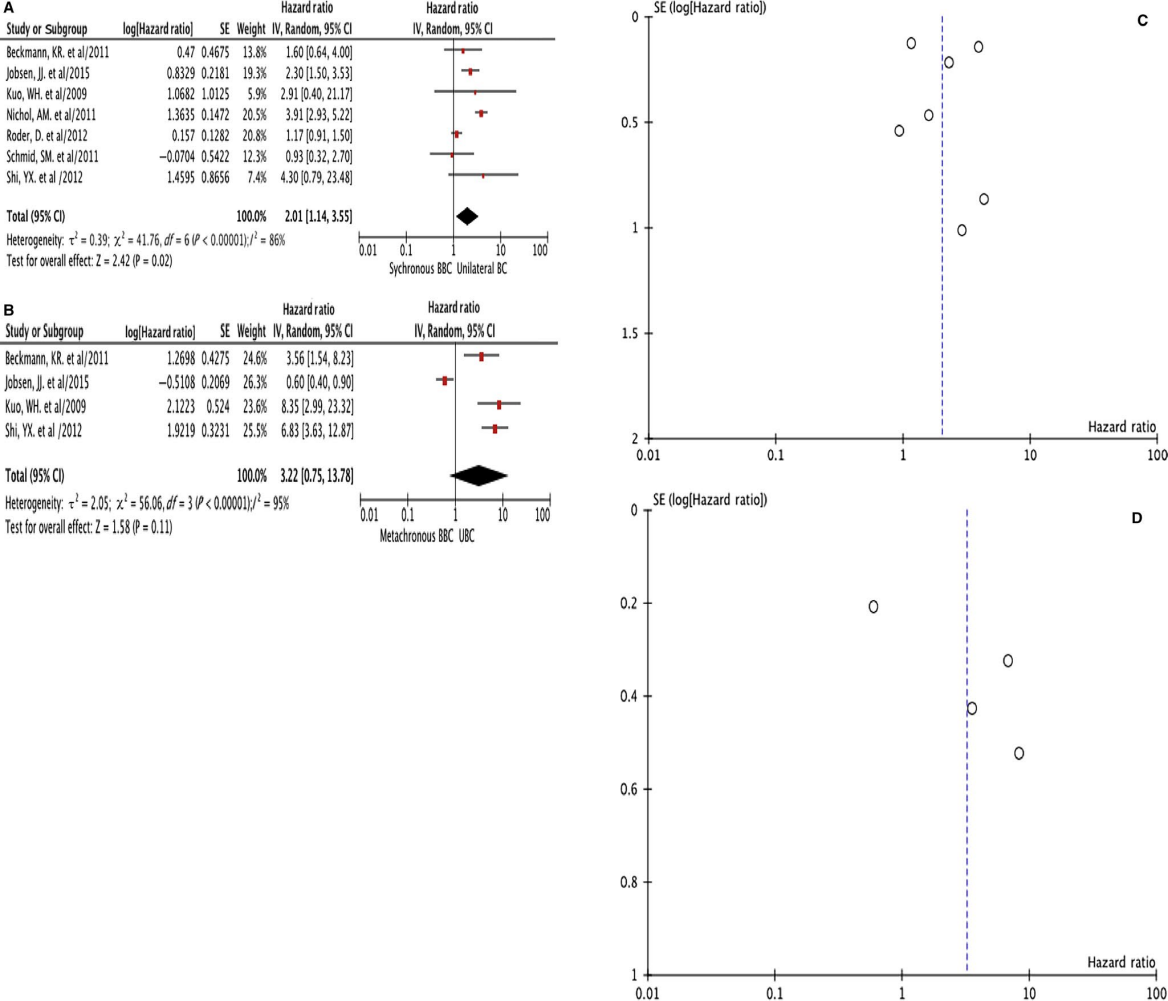
Hazard ratio (HR) of overall survival (OS) comparison of synchronous bilateral breast cancer (SBBC) vs unilateral breast cancer (UBC) forest plot (A); metachronous bilateral breast cancer (MBBC) vs UBC forest plot (B); SBBC vs UBC funnel plot (C) and MBBC vs UBC funnel plot (D)

Seven studies were included in the comparison of HR of OS between SBBC and UBC, including 1195 SBBC and 62 101 UBC (Figure 3A,C). The follow‐up time ranged from 4.6 to 10.2 years (median 6.6 years). The summary HR of SBBC vs UBC was 2.01 (95% CI = 1.14‐3.55). There was heterogeneity (I^2 ^= 86%, *P* < 0.00001) so random effects model was used. SBBC manifested worse OS than UBC.

Four studies were summarized in the analysis of HR of OS between MBBC and UBC, including 449 MBBC and 11 947 UBC (Figure 3B,D). The follow‐up time ranged from 4.6 to 9.2 years (median 5.8 years). The summary HR of MBBC vs UBC was 3.22 (95% CI = 0.75‐13.78). There was heterogeneity (I2 = 95%, *P* < 0.00001) so random effects model was used. MBBC showed similar or no better OS as UBC.

### 3.4 The comparison of HR of OS between SBBC vs MBBC

**Figure 4:**
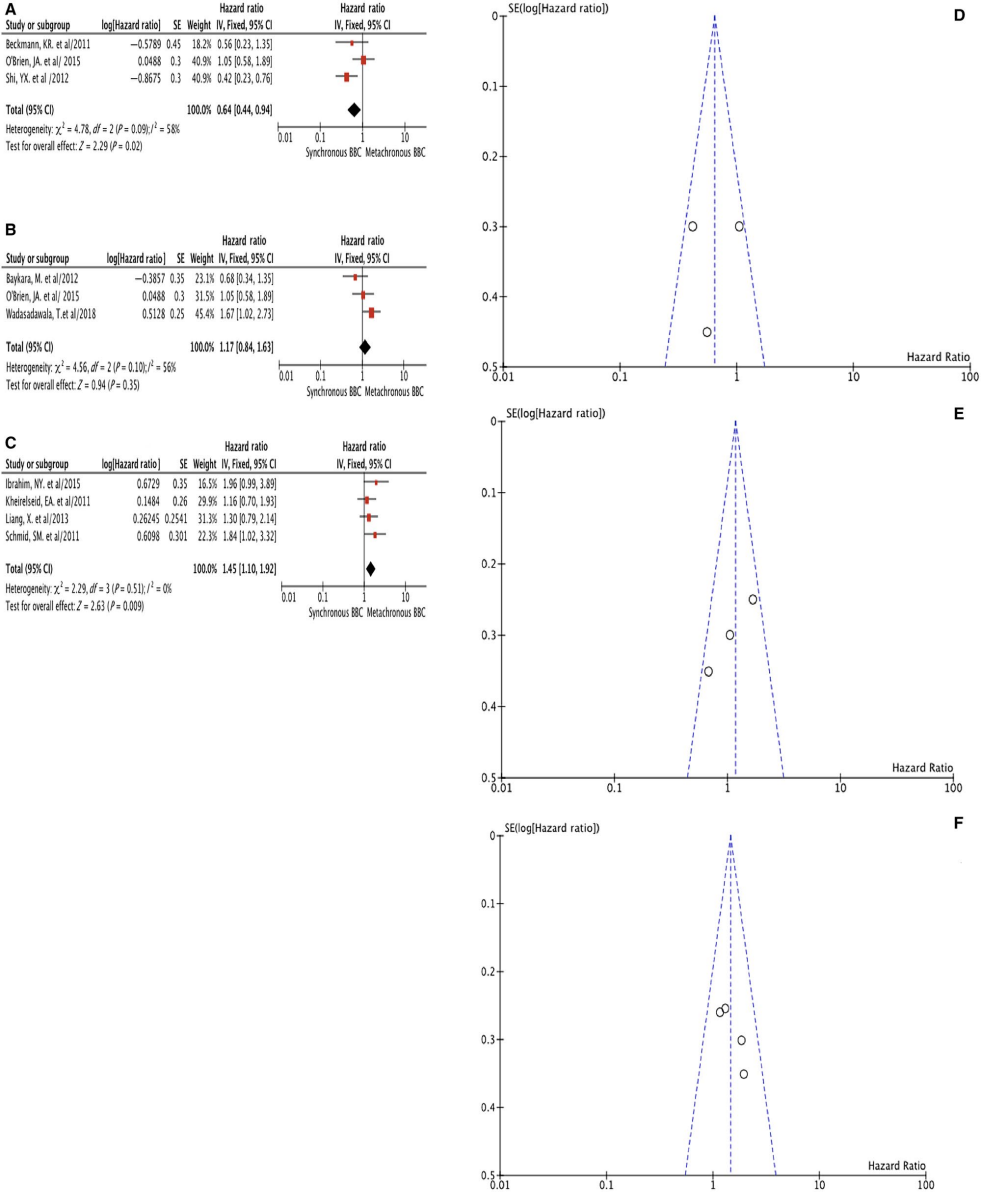
Hazard ratio (HR) of overall survival (OS) comparison of SBBC vs MBBC when 3, 6 and 12 months were used as interval time respectively: 3‐month interval time forest plot (A); 6‐month interval time forest plot (B); 12‐month interval time forest plot (C); 3‐month interval time funnel plot (D); 6‐month interval time funnel plot (E) and 12‐month interval time funnel plot (F)

Nine studies were included in the analyses the HR of OS between 747 SBBC vs 664 MBBC, with 4.58 years as median follow‐up time (2.92‐20 years) (Figure 4). These studies were divided into three groups based on the interval time to differentiate SBBC from MBBC (3, 6 and 12 months). The study from O'Brien et al^28^ used both 3 and 6 months as interval cut‐off (SBBC ≤ 3 months while MBBC > 6 months, Table 1), hence it was included in the meta‐analysis of both groups (Figure 4A,B). Three studies^7^, ^14^, ^28^ used 3 months as the interval, the summary HR of SBBC vs MBBC was 0.64 (95% CI = 0.44‐0.94) without heterogeneity (I^2 ^= 58%, *P* = 0.09) (Table 2, Figure 4A,D). Four studies^19^, ^22^, ^28^ adopted 6‐month as the interval time, and the summary HR was 1.17 (95% CI = 0.84‐1.63) without heterogeneity (I^2 ^= 56%, *P* = 0.10) (Table 2, Figure 4B,E). Twelve months was chosen as interval in the other four studies,^9^, ^13^, ^23^, ^24^ and the summary HR was 1.45 (95% CI = 1.10‐1.92) without heterogeneity (I^2^ = 0%, *P* = 0.51) (Table 2, Figure 4C,F). The HR of OS would change with the interval used. The longer the interval time used, the worse the survival of SBBC. When the interval was set to 6 months, SBBC and MBBC showed similar OS (Table 2).

**Table 2:** Hazard ratios of OS comparison of SBBC vs MBBC by different intervals

Interval time	Number of included study [reference]	HR and 95% CI of OS of SBBC vs MBBC
3 mo	3^7^, ^14^, ^28^	0.64 (0.44‐0.94)
6 mo	3^19^, ^22^, ^28^	1.17 (0.84‐1.63)
12 mo	4^9^, ^13^, ^23^, ^24^	1.45 (1.10‐1.92)

## 4 DISCUSSION

Owing to the growing awareness, prolonged lifetime and the modern screening methods, the incidence of BBC had been rising.^2^, ^20^ Evidence also suggested that incidence of SBBC had increased with age and by 40% during the 1970s.^2^ The annual risk of contralateral MBBC was ~0.5%, and would increase to 3% in BRCA1 or BRCA2 mutation carriers, making 10‐year risk up to 13%‐40%. Risk factors for contralateral BC included young age at first diagnosis of breast cancer and a family history of breast cancer.^6^, ^29^, ^30^ However, the majority of BBC could not be explained by BRCA carriership.^20^, ^31^ Interestingly, the risk of third primary cancers of non‐breast origin among women with previous BBC history would also increase, indicating that BBC might be genetically susceptible to develop cancer.^32^ Tumor profile of SBBC included correlations with age^2^, ^11^, ^33^ and lobular histology.^3^, ^7^, ^16^, ^18^ BBC demonstrated extensive inter‐tumoral and intra‐tumoral heterogeneity with pathogenic germline mutations including BRCA1 and TP53^34^ and a distinct miRNA profile with higher level of miR‐21, miR‐10b, and miR‐31.^35^ A small subset of contralateral BC was clonally related to metastatic dissemination from the index tumor regardless of whether the two tumors occurring as SBBC or MBBC.^36^ Thus a proportion of contralateral BC might actually be metastases instead of a new primary cancer, which asked for precise molecular differential diagnosis and individualized therapy for BBC patients.

There were controversies about whether adjuvant therapy for BBC should base on the higher risk tumor or the index tumor.^20^ Adjuvant chemotherapy might paradoxically both reduce the risk and worsen the prognosis of MBBC.^2^ Indeed, patients with BBC patients had a lower pathological complete remission (pCR) rate after receiving neoadjuvant chemotherapy and a lower DFS than UBC patients. The lower pCR rate after neoadjuvant chemotherapy in BBC might be due to the higher percentage of lobular carcinomas with more luminal histology compared to UBC.^37^

The result of our study was consistent with evidence that the prognosis of UBC was non‐inferior to that of BBC and that SBBC was an independent negative prognostic factor.^7^, ^8^, ^9^, ^10^, ^11^, ^12^, ^13^, ^14^, ^15^, ^20^ In this study, sensitivity analysis suggested that the follow‐up time might cause the heterogeneity among studies comparing BBC vs UBC. When we divided the 10 included studies into 3 groups according to the follow‐up time (<5, 5‐10 and > 10 years), all groups showed that BBC had a worse OS than UBC without any heterogeneity (Figure 2). With prolonged follow‐up time, some UBC might turn into BBC, however, BBC indicated worse prognosis compared to UBC regardless of the follow‐up time (Figure 2).

Most controversies existed in the comparison of survival between MBBC vs UBC and between SBBC vs MBBC. When ethnicity was taken into account, Asian women with SBBC tended to have an even lower 5‐year OS compared to those with MBBC, despite having seemingly biologically favorable ER/PR positive and Her2 negative tumors, which suggest that there may be more underlying their tumor biology and genetics.^38^ Studies also suggested that the second tumor developed after more than 5 years among MBBC gave rise to the improved survival^4^ whereas women with a BBC diagnosed more than 10 years after the first cancer had a prognosis similar to that of UBC.^2^ These results coincided with our findings that MBBC showed a prognosis similar or non‐superior to that of UBC (HR = 3.22, 95%CI: 0.75‐13.78, Figure 3B). Furthermore, with the multiple comparison performed with 3, 6, 12 months as interval time respectively, the dynamic trend of SBBC vs MBBC implied that the SBBC with 3‐12 months interval between two tumors had the worst survival (Table 2 and Figure 4). This subgroup of BBC patients often suffered from a secondary contralateral BC resistant to systemic adjuvant therapy such as chemo‐, targeted or endocrine therapy, which was usually administered during 3‐12 months after the diagnosis and surgery of the first tumor. Therefore, the survival difference between BBC and UBC was primarily due to the poorer prognosis of SBBC, and the worst survival of all BBC belonged to the subgroup of SBBC with 3‐12 months interval which developed under systemic adjuvant treatment. When this interval was set to 6 months, some of these BBCs with compromised prognosis were regarded as SBBC (within 3‐6 months interval) while the other BBCs with unfavorable survival taken as MBBC (within 6‐12 months interval), which made the difference between SBBC vs MBBC insignificant (Table 2 and Figure 4). Taken together, the results implied that the survival ordered from poor to favorable might be like: SBBC with 3‐12 months’ interval <SBBC<BBC<MBBC with 12 month's interval = UBC.

The strength of this meta‐analysis included: Firstly, only studies published within recent 10 years were included to ensure the patients received up‐to‐date BC treatment. In view of the period of the included studies (Table 1), only three studies dated back to early 1980s,^8^, ^24^, ^28^ when chemotherapy, radiation therapy and endocrine therapy had already been integrated into the comprehensive treatment of BC. Secondly, multiple comparisons were performed among all subgroups of BBC within the same study including BBC vs UBC, SBBC vs UBC, MBBC vs UBC and SBBC vs MBBC, which made the information complete. Thirdly, the comparison between SBBC vs MBBC were performed with several interval times, showing a dynamic trend of how the HR would change with the interval time. Last but not the least, the study population in this meta‐analysis was quite diversified in races and ethnicities including studies from five continents to ensure the result could be extrapolated to different ethnicities.

There were certain limitations of this study. Firstly, the included 2912 BBC and 72 302 UBC came from only 15 studies and there was considerable heterogeneity among them. And bias might be brought into analysis when prospective and retrospective studies were compared at the same time. The difference in follow‐up time between UBC and MBBC was unavoidable yet consequential, thus the retrospective studies might have flaws whereas the prospective and case‐control studies might be more balanced. Secondly, due to limited information on prognosis, only the OS was taken as survival endpoint, and there was little information on BC specific survival or disease‐free survival in this meta‐analysis. Thirdly, in the comparison of SBBC vs UBC and MBBC vs UBC, some SBBC in study A might be judged as MBBC according to the interval criteria in study B and C. However, this happened only to limited cases and would not change the results and conclusion. Fourthly, there was no information in terms of how the age, histology and genetic alterations might influence the survival of BBC. Treatment such as ipsilateral and contralateral re‐radiation, different chemotherapy regimen, changes in use of anti‐Her2 targeted agents and compliance of endocrine therapies as well as the molecular subtypes might also play their roles as confounders of survival.

## 5 CONCLUSION

BBC and SBBC both showed worse prognosis than UBC whereas MBBC presented non‐superior survival compared to UBC. As for SBBC and MBBC, various interval times indicated different prognosis profile. SBBC with interval of 3‐12 months indicated poor response and even resistance to adjuvant therapy, thus possessed the worst prognosis. When this interval was set to 6 months, SBBC and MBBC manifested similar survival.

## CONFLICT OF INTEREST

The authors declare that they have no competing interests.

## AUTHORS' CONTRIBUTIONS

Bo Pan and Ying Xu reviewed the literature and drafted the manuscript. Qiang Sun, Yi‐Dong Zhou and Bo Pan participated in the design of the study. Ying Xu and Chang‐Jun Wang performed the statistical analysis. Ru Yao, Huan‐Wen Wu, Qing‐Li Zhu, Feng Mao, Yan Lin, Song‐Jie Shen conceived of the study, and participated in its design and coordination and helped to draft the manuscript. All authors read and approved the final manuscript.

## Data Availability

Data sharing not applicable to this article as no datasets were generated or analysed during the current study.
